# Proteomic Approaches to Dissect Host SUMOylation during Innate Antiviral Immune Responses

**DOI:** 10.3390/v13030528

**Published:** 2021-03-23

**Authors:** Marie Lork, Gauthier Lieber, Benjamin G. Hale

**Affiliations:** Institute of Medical Virology, University of Zürich, 8057 Zürich, Switzerland; lork.marie@virology.uzh.ch (M.L.); lieber.gauthier@virology.uzh.ch (G.L.)

**Keywords:** SUMO, ubiquitin-like modification, proteomics, virus infection, influenza, interferon, innate immunity, ISG15, endogenous retroelements, TRIM28

## Abstract

SUMOylation is a highly dynamic ubiquitin-like post-translational modification that is essential for cells to respond to and resolve various genotoxic and proteotoxic stresses. Virus infections also constitute a considerable stress scenario for cells, and recent research has started to uncover the diverse roles of SUMOylation in regulating virus replication, not least by impacting antiviral defenses. Here, we review some of the key findings of this virus-host interplay, and discuss the increasingly important contribution that large-scale, unbiased, proteomic methodologies are making to discoveries in this field. We highlight the latest proteomic technologies that have been specifically developed to understand SUMOylation dynamics in response to cellular stresses, and comment on how these techniques might be best applied to dissect the biology of SUMOylation during innate immunity. Furthermore, we showcase a selection of studies that have already used SUMO proteomics to reveal novel aspects of host innate defense against viruses, such as functional cross-talk between SUMO proteins and other ubiquitin-like modifiers, viral antagonism of SUMO-modified antiviral restriction factors, and an infection-triggered SUMO-switch that releases endogenous retroelement RNAs to stimulate antiviral interferon responses. Future research in this area has the potential to provide new and diverse mechanistic insights into host immune defenses.

## 1. General Overview of SUMOylation and the SUMO Machinery

Since their discovery in 1996 [1], the small ubiquitin-like modifier (SUMO) proteins have emerged as key post-translational modifiers associated with crucial regulatory roles in the cell. SUMO proteins are small polypeptides of around 12 kDa that can be covalently conjugated to specific lysine residues on target proteins through a process called SUMOylation, thus altering target protein localization, stability, and/or interactions with other macromolecules. As such, SUMOylation plays an essential role in several cellular processes, including DNA damage repair responses, chromatin remodeling, transcriptional regulation, and signal transduction [2]. SUMO proteins are highly conserved across all eukaryotes, and to date, five genes encoding potential SUMO paralogs have been identified in humans. SUMO1, 2 and 3 are ubiquitously expressed, and have been the most extensively studied [2]. SUMO2 and 3 are very similar to one another, sharing 97% sequence identity, whereas SUMO1 shares only 47% identity with SUMO2/3 [3].

**Figure 1:**
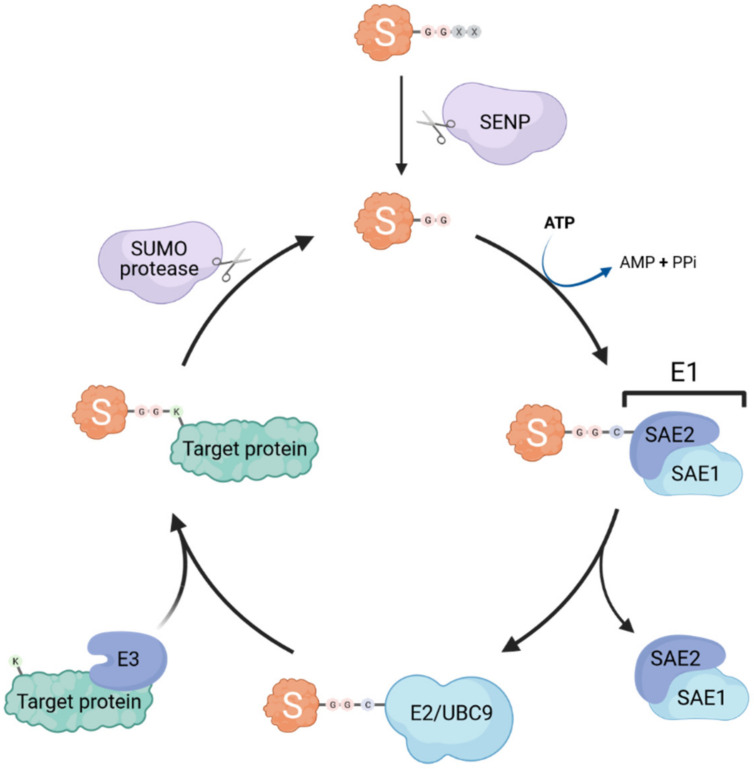
The SUMOylation machinery. Small ubiquitin-like modifiers (SUMOs; S in the cartoon) are covalently attached to lysine (K) residues in target proteins through the concerted action of dimeric E1 activating enzymes (SAE1/SAE2) and an E2 conjugating enzyme (UBC9). SUMOylation is usually aided by specific SUMO E3 ligases. SUMO specific proteases (e.g., SENP family members) can de-conjugate SUMO from its targets (known as deSUMOylation) and are also essential for the proteolytic maturation of SUMO precursors by cleaving off C-terminal residues to expose the di-glycine motif that is necessary for conjugation.

Similar to ubiquitination, SUMOylation occurs through the sequential action of E1 activating enzymes, E2 conjugating enzymes and E3 ligases (summarized in Figure 1). Interestingly, only a single E1 (heterodimeric SAE1/SAE2) and E2 (UBC9) exist, whereas several SUMO E3 ligases have been identified (e.g., some TRIM proteins, RANBP2, and PIAS proteins) [4,5,6]. Thus, it is likely that the E3 ligases are the main actors conferring substrate specificity and SUMO paralog selection [7]. SUMO proteins are expressed as precursors that require a proteolytic maturation step before SUMOylation can occur: sentrin-specific proteases (SENPs) cleave the SUMO precursor’s C-terminal tail to expose the diglycine motif essential for subsequent conjugation [8,9]. The E1 activating enzyme then forms a thioester bond between its catalytic cysteine and the terminal glycine of mature SUMO in an ATP-dependent reaction, before transferring the SUMO adenylate to the E2 SUMO-conjugating enzyme UBC9 [10]. The SUMO protein is then further transferred by UBC9 to an acceptor lysine of the substrate, forming an isopeptide bond between the carboxyl group of the C-terminal glycine residue of SUMO and the ε-amino group of the lysine side chain. The target lysine is typically located in a specific consensus motif ψKxD/E (where ψ is a hydrophobic residue, x is any amino acid), that has been identified in more than half of all known SUMO target proteins [11]. This process is usually facilitated by SUMO E3 ligases that catalyze the transfer of SUMO from UBC9 onto the target, however, E3-independent SUMOylation has also been observed [12].

Notably, SUMO2 and SUMO3 contain internal lysines that can serve as SUMO acceptor sites themselves, thus permitting the formation of polySUMO chains [2]. The formation of SUMO chains is promoted by specific SUMO E3 ligases that also carry SUMO E4 elongase activity, such as ZNF451 family members [13]. SUMO chains increase the functional diversity of SUMO modification, as many consequences of SUMOylation include the recruitment of specific SUMO-interacting motif (SIM)-containing proteins, which thereby nucleate multi-molecular signaling complexes, or trigger changes in localization or protein stability [14]. For example, SIM-containing SUMO-targeted ubiquitin ligases (STUbL) can be recruited to SUMOylated targets and result in ubiquitination of SUMO chain-containing proteins, thus marking them for proteasome-mediated degradation [15].

Importantly, the SUMOylation process is also reversible, and counteracted by various SUMO-specific proteases, which are divided into three classes based on the fold of their respective catalytic domains: ubiquitin-like protease/sentrin-specific protease (Ulp/SENP) family, the deSUMOylating isopeptidase (Desi) family, and ubiquitin-specific peptidase-like protein 1 (USPL1) [16]. These proteases are able to cleave the isopeptide bond between SUMO and its substrates. Thus, SUMOylation is a highly dynamic mechanism by which the cell can quickly regulate numerous functional pathways, most notably under various stress conditions, without altering protein synthesis [17].

## 2. SUMOylation Is a Key Regulator of Innate Antiviral Immunity

While the contributions of SUMOylation to cellular DNA damage repair responses, chromatin remodeling, and transcriptional regulation have been well established, there is emerging evidence that SUMO modifications also play critical roles in the cell’s ability to respond to, and resolve, virus infections. Partly, this is due to the tight interplay between virus and host, and the necessary viral dependencies on many of the cellular processes regulated by SUMO, which viruses have exquisitely evolved to utilize or to perturb (comprehensively reviewed previously in [18]). However, it is now clear that SUMOylation may exert additional, specific, functions in innate antiviral immunity, and in particular the host interferon (IFN) response. The IFN system consists of a cellular mechanism to ‘sense’ infection (usually by detection of viral nucleic acids), followed by a cytokine-based (IFN) signaling cascade that confers protection to cells via transcriptional upregulation of several hundred antiviral IFN-stimulated genes (ISGs) (for several excellent and comprehensive reviews, please see [19,20,21]).

The production and signaling of IFNs must be tightly controlled in order to prevent overexuberant (auto-) immune reactions that can otherwise lead to severe disease consequences [22]. Mechanistically, this regulation is thought to primarily involve ‘fine-tuning’ of individual protein functions in the relevant signaling pathways by post-translational modifications [23], among them SUMOylation. Indeed, many individual targeted studies have reported dynamic regulatory SUMOylation events on key factors involved in almost all stages of the IFN response, including sensing virus infections (RIG-I, MDA5, cGAS) [24,25,26], signaling leading to IFN transcription (MAVS, STING, IRF3/7) [25,26,27,28], signaling leading to ISG transcription (STAT1) [29], or activity of ISGs directly (PML, PKR, SAMHD1, MxA/B, ADAR1) [30,31]. In addition, several corresponding SUMO ligases, such as some TRIM family members, and SENPs have been described to be involved in innate antiviral immune responses [24,25,32]. Thus, depending upon the context and the factors involved, the SUMOylation system (like many other post-translational modification systems) seems to have either positive or negative effects on IFN-mediated control of virus infection. These SUMO-dependent mechanisms have recently been reviewed exhaustively elsewhere [30], and we will not re-evaluate these findings in depth here.

We note that many of the reported SUMO-modified IFN-related host factors have not been identified as such in any of the large-scale unbiased SUMO proteomic experiments published to date [33,34,35,36,37,38], with the key exceptions of examples such as STAT1, PML and SAMHD1, as well as ADAR and PIAS1-4 [33,34,35,36,37,38]. Thus, there seems to be an unresolved discrepancy between results obtained from individual targeted studies and results obtained from global proteomic screens. While this may reflect the unavoidable intrinsic limitations of overexpression-based assays (used by our laboratory and others in both targeted SUMO studies and SUMO proteomic screens), sensitivity issues with mass spectrometry (MS) for certain peptides/proteins, or poor availability of specific reagents for some SUMO- or IFN- related factors, it still raises important unanswered questions as to the precise roles of SUMOylation in the IFN response, and how to address these reproducibly and informatively.

Nevertheless, we draw the reader’s attention to results from two critical studies that we find particularly important in the context of SUMOylation and IFN. Firstly, using a conditional *Ubc9* knock-out system in mice, Decque et al. revealed that loss of murine *Ubc9* results in reduced levels of global SUMOylation and a spontaneous IFN response that is characterized by constitutive upregulation of *Ifnb* expression and a consequently enhanced ISG transcriptional signature, as shown by genome-wide microarray analysis [39]. This indicated that SUMOylation seems to specifically regulate certain innate immune loci rather than exerting general effects on transcription or the epigenetic landscape. In addition, diminished SUMOylation caused by loss of *Ubc9* led to enhanced innate antiviral immune responses following stimulation of viral sensing pathways, and enhanced resistance to viral infection [39]. Somewhat similar results were obtained by Crowl et al., who also found that knock-out of the SUMO conjugation machinery (e.g., *SAE1*, *SAE2* or *UBC9*) in human cells led to increased *IFNB1* expression and transcription of an ISG signature [40]. Finer pathway mapping further revealed that SUMO2 and SUMO3 are redundant regulators of the IFN response, with combined depletion of SUMO2 and SUMO3 resulting in increased *IFNB1* and ISG expression [40]. These complementary, and independent, studies provide convincing evidence for the key role of SUMOylation in regulating innate antiviral IFN responses. The mechanistic basis for SUMO2/3 acting to restrain *IFNB1* expression has yet to be fully resolved, but SUMO2/3 conjugation (surprisingly in monoSUMOylation form) seems to be a key mechanism [40]. Given that spontaneous *IFNB1* expression in SUMO2/3-deficient human cells appears to be independent of canonical IFN induction pathway components (such as STING, MAVS, the TBK1-like kinases, IRF3, and IRF7), it has been speculated that direct SUMO2/3 conjugation to other IFN-related transcription factors, such as IRF1 or IRF5, may normally act to restrain *IFNB1* transcription [40,41]. Indeed, SUMO2 has been identified at genomic enhancer regions of the *Ifnb1* locus in murine cells [39], but the relevant host proteins targeted by SUMO in this context remain to be identified.

A key challenge in this field remains the ability to identify the specific cellular targets and consequences of SUMOylation, particularly with regards host response changes that occur during virus infection as part of innate antiviral immunity. Without doubt, future unbiased SUMO proteomic efforts will be essential to dissect these pathways further, but such approaches have not yet been employed as widely as in the general cell biology and DNA damage response fields [38]. In the following sections, we will therefore discuss different technologies that have been developed to investigate system-wide changes in cellular SUMOylation, and how these might be applied to studying SUMOylation in the context of viral infection and innate antiviral immunity. We will also summarize some key findings from the few SUMO proteomic studies that have already been performed in this field, and highlight the new and unexpected findings that they have revealed.

## 3. Strategies and Technologies to Identify SUMOylated Proteins

**Figure 2:**
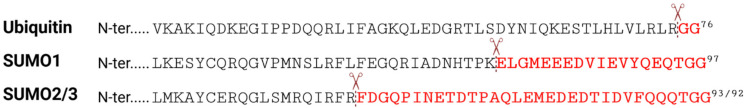
The C-terminal amino-acid sequences of ubiquitin and SUMO paralogs. Scissors indicate trypsin cleavage sites. Amino acid remnants that remain on UBL-modified peptides after tryptic digest are highlighted in red.

Given the contribution of SUMOylation to the regulation of innate antiviral immune responses, there has been a substantial research interest in identifying, and functionally characterizing, protein targets that change in SUMO-modification status in response to the stress of virus infection. However, as discussed above, most studies to date have mainly relied on targeted analysis of specific known proteins of interest that had already been implicated in certain pathways, and their SUMOylation status was investigated by standard techniques such as overexpression (sometimes together with components of the SUMO machinery), immunoprecipitation, and western blotting. These methods usually limit analyses to only a single protein at a time, and thus it could be of great benefit to adopt less biased approaches that can identify SUMOylated proteins (preferably expressed under endogenous conditions) at a system-wide scale. MS-based proteomics allow for direct mapping and quantification of numerous post-translational modifications (PTMs), and covalent modifications often result in mass changes to modified peptides that can be directly detected [42]. This enables the identification of modified proteins and mapping of the specific modified residue, as well as determination of changes in abundance if methods such as Label Free Quantification (LFQ) or Stable Isotope Labeling with Amino Acids in Cell Culture (SILAC) are employed [43]. Nevertheless, efficient large-scale analysis of specific PTMs requires prior enrichment of modified peptides. For example, tryptic digest of ubiquitinated proteins results in peptides containing a diglycine (GG) remnant from the two C-terminal glycine residues of the conjugated ubiquitin on the modified lysine (K) (Figure 2). A specific antibody raised against this K-ε-GG motif was generated to enrich for ubiquitin-modified peptides prior to MS, creating a highly efficient enrichment strategy that is widely applied to investigate endogenous ubiquitination [44]. In contrast, MS-based identification of SUMO substrates and SUMOylation sites has proved a lot more challenging. Tryptic digest of SUMOylated proteins leaves a larger signature tag on modified peptides (19 or 32 amino acids for mammalian SUMO1 or SUMO2/3, respectively (Figure 2)) that interferes with their identification due to complex MS/MS fragmentation patterns [11]. In addition, many proteins that can be SUMO-modified appear to have a very low abundance in the cell, and only a small percentage of these proteins are modified at any one time [45]. Furthermore, the deSUMOylation activity of SUMO proteases is rather high in cell lysates, resulting in rapid loss of SUMOylation unless preventative steps are taken [46]. These peculiarities of the SUMO system have sometimes hampered general efforts to identify SUMOylated proteins (as compared with ubiquitinated proteins), and therefore new methodologies and procedures have had to be developed, although each of these technologies also has caveats.

### 3.1. Epitope-Tagging to Assist Purification of SUMO-Modified Proteins

**Figure 3:**
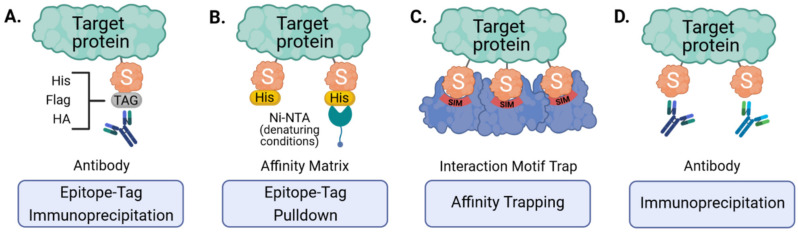
Strategies for the enrichment of SUMOylated target proteins. Ectopically expressed, epitope-tagged SUMO can be purified using tag-specific antibodies (**A**) or affinity matrices that bind specific tags (e.g., His6-tag and Ni-NTA under denaturing conditions) (**B**). Endogenous SUMO can also be enriched by engineered SUMO-traps that consist of multiple SUMO-interacting motifs (SIMs) immobilized onto an affinity matrix (**C**) or by immunoaffinity purification using SUMO-specific antibodies (**D**).

To facilitate enrichment of SUMO conjugates prior to trypsin digest and MS, several groups have made use of ectopically expressed epitope-tagged SUMOs (Figure 3A,B). These affinity tags commonly include polyhistidine- (His6), Flag-, HA- or Tandem Affinity Purification (TAP)- tags that are small enough to permit SUMO conjugation and function, but allow for efficient purification at high yields [47,48,49,50,51,52]. High stringency purification of tagged SUMOs is achieved by the strong interaction between certain tags and their corresponding affinity matrices (e.g., His and Ni-NTA) that can allow for denaturing cell lysis and harsh washing conditions, and is essential to inactivate SUMO proteases and to dissociate proteins interacting non-covalently [51,52]. Such methods can be important for minimizing the likelihood of detecting false-positive SUMO substrates, and are particularly powerful when combined with ‘slice-by-slice’ analyses, where purified proteins are separated by SDS-PAGE alongside corresponding total cell lysates, and the resulting MS data from individual gel slices are interpreted to validate SUMO-associated mass shifts [33,47,49]. Ectopic expression of epitope-tagged SUMOs followed by purification and MS has therefore proven a useful tool, as it has been successful in identifying more than a thousand candidate SUMO targets, although a major caveat is that it is unable to give information on the specific site of SUMO modification [38], which is critical for follow-up studies. Our laboratory has also found that this method may not be ideal for identifying SUMO paralog-specific substrates, as experiments using overexpressed TAP-tagged SUMO1 or SUMO2 unexpectedly yielded similar SUMO target profiles [33]. Furthermore, and with respect to innate antiviral immunity, recent studies have also shown that ectopic expression of SUMO3 results in lower STAT1 activation in response to IFNα [35], providing yet more evidence that SUMO overexpression may unintentionally bias results. In addition, most of these techniques only allow analysis of one SUMO paralog at a time. SUMO overexpression also excludes the investigation of endogenous SUMOylation responses, particularly in in vivo tissues. Therefore, in an attempt to study SUMOylation in a more physiological way, genetic engineering has been used to generate knock-in mice with epitope-tagged SUMO [50]. The advantage is that SUMO is expressed at close-to-endogenous levels, which lowers the risk of overexpression artefacts, and SUMOylation can be studied in different tissues. Thus, while epitope tagging and denaturing purification is no doubt an important technology for identifying SUMOylated proteins, future applications, particularly in cell models, should consider the use of gene-editing strategies to tag endogenous SUMOs and thereby ensure appropriate expression levels [53].

### 3.2. Re-Engineering SUMO to Identify Sites of Modification on Target Proteins

**Figure 4:**
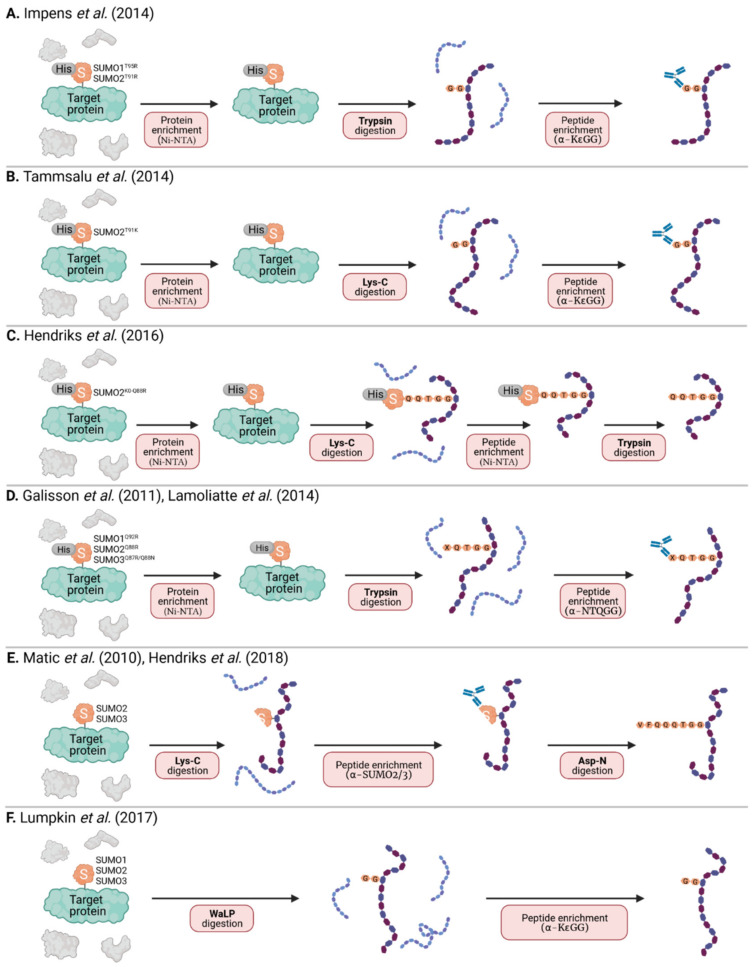
MS-based strategies to identify specific SUMOylation sites. (**A**) His6-SUMO1-T95R or HisSUMO2-T91R modified proteins are purified by Ni-NTA, digested with trypsin, and peptides containing the di-glycine remnant are enriched using a specific α-KεGG antibody. (**B**) His6-SUMO2 T91K modified proteins are purified by Ni-NTA, digested with Lys-C, and peptides containing the diglycine remnant are enriched using a specific α-KεGG antibody. (**C**) Proteins modified with His10-SUMO2-K0-Q88R (K0 = all K residues mutated to R) are purified by Ni-NTA and digested with Lys-C. Peptides containing the intact His10-SUMO modification are then enriched by a second Ni-NTA purification step before trypsin digest. (**D**) His6-SUMO1 Q92R, His6-SUMO2 Q88R and His6-SUMO3 QQ87/88RN modified proteins are purified by Ni-NTA, digested with trypsin, and peptides containing a (X)QTGG remnant are enriched using a specific α-NQTGG antibody. (**E**) Target proteins modified with endogenous SUMO are digested with Lys-C. Peptides containing a SUMO fragment (with the intact SUMO2/3 antibody epitope) are then immunoaffinity-purified before a second digestion step with Asp-N. (**F**) Target proteins modified with endogenous SUMO are digested with the endoproteinase WaLP, and peptides containing the di-glycine remnant are enriched using a specific α-KεGG antibody. For all methods (**A**–**F**), enriched peptides are analyzed by mass spectrometry (MS) for identification and quantification.

As stated above, tryptic digest (most commonly used in MS) of SUMOylated proteins leaves a large signature tag on modified peptides that interferes with their identification. This is not an issue with ubiquitin due to the presence of an arginine residue immediately preceding its C-terminal di-glycine motif, forming a trypsin cleavage site that leaves only a small remnant on modified peptides. In SUMO paralogs, the C-terminal di-glycine sequence is preceded by threonine, and there is no other tryptic cleavage site near its C-terminus (Figure 2). To overcome this problem, strategic mutations were introduced into the SUMO sequence to result in more convenient tryptic cleavage fragments. Impens et al. generated His6-tagged versions of SUMO1 and SUMO2 with an arginine residue immediately before the C-terminal di-glycine motif (SUMO1 T95R and SUMO2 T91R) imitating the sequence of human ubiquitin (Figure 2 and Figure 4A) [54]. Peptides containing this di-glycine remnant after trypsin digest could then be purified using the K-ε-GG immunoaffinity enrichment pipeline established for ubiquitinated proteins, and processed to identify sites of diglycine modification on target proteins. Unfortunately, this approach does not differentiate between the tryptic di-glycine remnant derived from the mutant SUMO and the diglycine remnant from other ubiquitin-like modifiers (UBLs), including ubiquitin, ISG15 and NEDD8. Thus, this method still requires expression of epitope-tagged SUMO, and the prior purification of SUMO conjugates before preparation of tryptic peptides.

A further caveat arises when one considers the possibility that a particular SUMO-modified protein may also be ubiquitinated, therefore this method may still map both SUMOylation and ubiquitination sites in some proteins. To overcome this, Tammsalu et al. took advantage of the enzymatic specificity of the endoproteinase, Lys-C, which only cleaves at the C-terminal side of lysines unlike trypsin that also cleaves at the C-terminal side of arginine residues [55]. Thus, Tammsalu et al. substituted the threonine near the SUMO2 C-terminus to lysine (His6-SUMO2-T91K) generating a novel Lys-C cleavage site that also results in the di-glycine remnant on modified peptides, and which can be recognized by the K-ε-GG antibody for purification prior to MS (Figure 4B). Importantly, in this scenario, Lys-C (unlike trypsin) would not cleave other UBLs to reveal di-glycine remnants, thus ensuring that subsequent MS of purified peptides reveals di-glycine modification sites specific to SUMO, and not other UBLs [55]. For clarification, Tammsalu et al. (as well as some of the subsequent authors) based their studies on the SUMO amino acid sequences described by Tatham et al. [56], which were cloned from HeLa cells and differ from the SUMO sequence found on Uniprot by a few amino acids. Therefore, Tammsalu et al. refer to the SUMO2 mutant they generated as T90K [55].

Overall, methods to provide site-specific mapping of SUMO conjugates in target proteins creates a very rich additional layer of information as compared to only identifying SUMO target proteins. This information immediately opens up the possibility of functional experiments where specific SUMO modification sites can be altered in target proteins to assess mechanistic consequences.

In a further development of the SUMO mutagenesis strategies, Matic and colleagues used a His6-tagged SUMO2 mutant in which all lysines were replaced by arginines (K0: making it resistant to cleavage by the protease Lys-C), and introduced additional arginines at positions 91 (T91R) or 88 (Q88R), the positions that correspond to arginines in ubiquitin or the yeast SUMO protein, Smt3 [11,57]. After Lys-C digest of total protein lysates, peptides covalently bound to the intact His6-SUMO2 were purified by Ni-NTA via the His6-Tag, and digested with trypsin to leave either a diglycine (His6-SUMO2 K0 T91R) or QQTGG (His6-SUMO2 K0 Q88R) SUMO signature peptide that is suitable for MS analysis. This K0 method was further optimized using a two-step purification strategy, where primary Ni-NTA purification of His10-SUMO2 K0 Q88R, which enriches for His10-SUMO conjugated proteins, is followed by Lys-C digest and a second Ni-NTA purification step to enrich SUMOylated peptides prior to trypsin digest and MS (Figure 4C) [58]. One caveat of the K0 technique is that the absence of lysines precludes the formation of polySUMO chains. Galisson et al. generated mutants of the three SUMO paralogs with an N-terminal His6 tag and a tryptic cleavage site near the C-terminus (His6-SUMO1 Q92R, His6-SUMO2 Q88R and His6-SUMO3 QQ87/88RN) resulting in a unique five amino-acid SUMO fragment (EQTGG for SUMO1, QQTGG for SUMO2, NQTGG for SUMO3) on modified lysines upon tryptic digestion (Figure 4D) [59]. The short amino-acid SUMO remnant facilitates the identification of SUMOylated peptides by conventional database search engines and enables the distinction of individual SUMO paralogs by mass-specific signature fragment ions. Furthermore, Lamoliatte et al. generated a highly selective monoclonal antibody (α-NQTGG) that specifically recognizes peptides containing either of these SUMO remnants, enabling further immunoaffinity enrichment analogous to the K-ε-GG antibody used for ubiquitin-remnant enrichment (Figure 4D) [60]. Thus far, the K0 method has proven to be one of the most efficient strategies allowing for routine identification of more than 1000 SUMO2 sites under standard conditions [38]. However, given that this system is limited to mono-SUMO2 modification, the approach developed by Galisson et al. and Lamoliatte et al., which features all the strengths of ubiquitin remnant profiling, might become increasingly popular.

### 3.3. Purifying and Identifying Endogenous SUMOylated Proteins

The above-described MS-based proteomic approaches have enabled effective mapping of SUMOylation sites. However, all require expression of exogenous SUMOs carrying epitope tags or mutations in the SUMO sequence, which might result in unnatural SUMO conjugation and potentially detrimental consequences for signal transduction. To address this, several techniques have now been developed that are directed towards the identification of endogenous SUMOylated proteins. In analogy to tandem-repeated ubiquitin-binding entities (TUBEs), containing four tandem ubiquitin binding domains connected by flexible linkers and fused to an affinity tag [61], the high affinity interaction between SUMO and SIMs can be exploited to isolate potential SUMO targets (Figure 3C). A fragment of RNF4 (residues 32–133) containing its four SIM domains immobilized onto an affinity matrix was used to efficiently purify endogenous polySUMO2/3 chains and attached proteins [62]. To increase SUMO-binding capacity, another group generated SUMO-traps (SUBEs: SUMO binding entities) containing four tandem SIM2-SIM3 motifs from RNF4 fused to a GST or biotin tag [63,64]. One drawback of using these SUMO traps is the limitation to polySUMOylation, as generally monoSUMOylated substrates are not bound by these matrices with high affinity. Furthermore, high-stringency or denaturing conditions cannot be used to limit the co-purification of non-covalently bound proteins, as these conditions could disrupt the SUMO-SIM interactions.

Immunoprecipitation with specific monoclonal antibodies recognizing endogenous SUMO1 or SUMO2/3 has also been used for efficient enrichment and identification of endogenous SUMO targets in mammalian cells and complex tissues (Figure 3D) [65]. To minimize non-specific interactions, proteins can be selectively eluted using minimal epitope-spanning peptides. Still, the use of antibodies for enrichment provides relatively low yields, and also requires mild buffer conditions resulting in a high amount of background and/or the co-purification of non-covalently bound proteins. Furthermore, although these ‘endogenous’ approaches have led to the identification of a few hundred putative SUMO target proteins, no SUMOylation sites on these proteins have been reported, as the tryptic remnants of SUMO modification are not suitable for MS-based identification (see above and Figure 2). To overcome this issue, and to enable the enrichment of SUMOylated peptides, this antibody–based affinity purification method was revised and further optimized by Hendriks et al. (Figure 4E) [66]. Total cell lysates were first digested with Lys-C, which leaves intact a specific antibody epitope of SUMO2/3, thus allowing for efficient antibody-based purification of peptides containing the remaining SUMO2/3-fragment. To generate smaller peptides suitable for MS, a second digest with the endoproteinase Asp-N enzyme is then performed, which cleaves after aspartic acid residues. The resulting peptides contain an eight amino acid stretch (VFQQQTGG) covalently attached to the modified lysine residue of a peptide. Using this approach, roughly 15,000 unique endogenous SUMO2/3 sites were identified in cultured cells [66]. As this technique does not require ectopic expression or genetic manipulation, it has also been utilized to analyze in vivo SUMOylation, identifying almost 2000 SUMO2/3 sites across several mouse organs.

Also aiming at the identification of endogenous SUMO sites at a system-wide scale, Lumpkin et al. made use of a wild-type α-lytic protease (WaLP), which has a relatively relaxed specificity, but was shown to preferentially cleave following threonine residues, and rarely after arginine (Figure 4F) [67]. Thus, WaLP cleaves all SUMO paralogs at their C-terminal TGG sequences, resulting in a SUMO-remnant KGG on SUMO-modified peptides. These peptides are suitable for enrichment by the K-ε-GG-affinity purification workflow already described for ubiquitin profiling [44], and because the samples are generated with WaLP, they should contain few ubiquitin-derived GG remnant peptides. Strikingly, with this technique, one sample can be analyzed for ubiquitinated and SUMOylated targets in parallel by splitting the sample into two fractions and subjecting each to either trypsin or WaLP digestion. One shortcoming, however, is that WaLP can also cleave after leucine (and to some extent after isoleucine) and hence does not exclude the possible detection of FAT10ylated and FUB1ylated proteins.

### 3.4. Purifying and Identifying Interactors of SUMOylated Proteins

**Figure 5:**
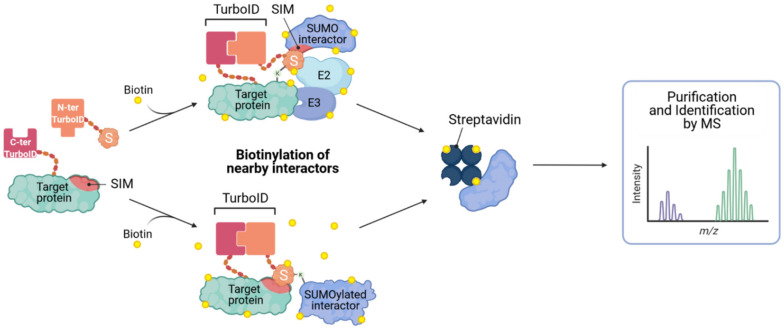
Identification of SUMO-interacting proteins by SUMO-ID. The N-terminal Split-TurboID construct is fused to the SUMO of interest, and the C-terminal fragment is fused to a potentially SUMOylated (or SIM-containing) target. Target SUMOylation, or SUMO-SIM interaction, results in reconstitution of the Split-TurboID and permits biotin labeling of proximal proteins. Biotinylated proteins can then be purified on streptavidin matrices and analyzed by mass spectrometry (MS). Concept developed in [68].

While most of the efforts described above focus on the identification of SUMO substrates and specific SUMOylation sites, it is clear that SUMO-dependent interactions are incredibly important in functionally ‘interpreting’ the changes in SUMOylation status of target proteins, either by affecting their cellular redistribution, complex formation, and/or stability. Thus, being able to identify SUMO-dependent interactions should also be a future goal of studies to better understand SUMOylation in the context of innate antiviral responses. As such, Barroso-Gomila et al. have developed a novel technology to identify SUMO-dependent interactors, termed SUMO-ID [68]. They devised a SUMO-centric approach that combines biotin proximity labeling by TurboID [69] with protein-fragment complementation (Split-TurboID), such that biotinylation is dependent on the proximity of the two fusion partners: one fragment of the Split-TurboID is fused to SUMO, and the other to a putative SUMOylated target (Figure 5). Target SUMOylation (or SUMO-SIM interaction) brings both fragments together, resulting in reconstitution of the TurboID enzyme, and allowing specific biotinylation of interactors, and other proximal proteins, in a SUMO-dependent manner. Biotin labeled proteins can then be enriched by streptavidin purification and identified by MS. This new method could prove to be a powerful tool for the identification of specific SUMO-dependent interactors of certain target proteins, and has the potential to allow functional discovery of SUMO enzymatic machinery or other ‘interpreters’ of SUMO modification.

In summary, several different methodologies have been developed over the years to identify SUMO-modified proteins and to map specific sites of SUMOylation. These techniques can be, and have been, applied in unbiased, system-wide approaches to understand how the SUMOylation status of proteins changes with certain stress conditions, and thus all of these technologies could similarly be applied to understand how host cells respond to infection. There is clearly an experimental balance to be made between strategies that permit robust and efficient purification of SUMO targets, the reliable determination of SUMOylation sites (which so far relies on ectopic expression of tagged, and sometimes mutant, constructs) and the unavoidable potential for ensuing artefacts. These problems may be overcome if the field adopts genome-editing strategies to tag endogenous genes, or uses purification methods (which are currently lower yield) that can enrich fully endogenous SUMOylated material.

## 4. Recent Proteomic Insights into Host SUMOylation and Innate Antiviral Immunity

While the methods and technical advances outlined in the preceding section will no doubt be instrumental in improving future MS-based proteomic approaches to investigate innate antiviral immunity, we note that several studies have already applied some of these techniques to this topic. Below, we briefly discuss what these large-scale, unbiased, proteomic studies have already taught the field about SUMOylation and innate immunity.

### 4.1. SUMO Proteomics during the IFN Response

Several studies have shown by western blotting that IFNα or IFNγ treatment induces an increase in both conjugated, as well as unconjugated, SUMO1 and SUMO2/3 in different cell lines and primary cells from mice and humans [35,70]. The SUMO upregulation is specific to the protein level, as IFN stimulation did not affect the abundance of SUMO transcripts [70,71]. SUMO availability in response to IFN stimulation was proposed to be regulated by a miRNA-based mechanism involving the Lin28/let-7 axis, and the increased SUMOylation contributed to the antiviral effects of IFNα against Herpes Simplex Virus type 1 (HSV-1) and Human Immunodeficiency Virus type 1 (HIV-1) [70].

The Chelbi-Alix group set out to identify specific IFNα–induced changes to SUMOylation using quantitative proteomics [34,35]. They used HEK293 cells stably expressing His6-SUMO3-Q87R-Q88N (see Section 3.2 and Figure 4D) [59] grown in SILAC medium, and treated them with IFNα for short (0.75 h) or long (16 h) periods of time, while mock treated cells were used as a negative control [35]. Cytoplasmic and nuclear protein extracts were subjected to Ni-NTA purification to enrich SUMOylated proteins and, following tryptic digest, SUMOylated peptides were further purified using a specific α-NQTGG antibody and identified by MS analysis. This protocol allowed for the specific enrichment and identification of peptides containing SUMO3-remnant–modified lysines, and a total of 558 SUMO3 sites were identified on 370 proteins, a considerable proportion of which were regulated by IFNα (172 out of the 558 SUMO3 sites). Among the IFNα-regulated SUMO3 substrates were several regulators of IFN production and signaling (including PML and STAT1), as well as proteins from the SUMOylation machinery (UBC9, SUMO proteins, PIAS family members, TRIM28, and PML). Further characterization revealed that the IFN-induced increase in global SUMOylation depended on PML, and that PML aids the recruitment of UBC9 to PML nuclear bodies, which the authors suggest promotes this enhancement of SUMOylation.

The above study showed that SUMO proteomics could be applied to the IFN system, and has produced a new, useful resource of potential SUMO3 substrates (and modification sites) with which to functionally dissect SUMO3 contributions to innate antiviral immunity. However, it was shown that ectopic expression of SUMO3 decreased IFNα-induced STAT1 phosphorylation [35], highlighting that SUMO3 overexpression might result in unnatural SUMO-conjugation that alters signal transduction. It will therefore be valuable in future studies to ascertain whether the same proteins are differentially SUMO-modified under conditions where SUMO3 is not overexpressed or mutated, as well as to delineate the sub-proteomes of SUMO1 and SUMO2, and to understand how these may differ from SUMO3. In this regard, a subsequent total cell proteomics study by the same group has shown that ectopic expression of SUMO3 in HeLa cells leads to a change in abundance of over 1000 proteins, both in the presence and absence of IFNα treatment, but without affecting RNA levels [34]. Strikingly, several ISG products were stabilized by SUMO3 expression (e.g., STAT1, IFIT family members, OAS3, PML, and GBP1), and some of them were already stabilized in cells that had not been treated with IFNα (e.g., SAMHD1, IFIT3, PKR). Interestingly, SUMO3 expression also stabilized components of the ISG15-conjugation machinery (HERC5, TRIM25), and the authors found that SUMO3 expression led to enhanced global cellular levels of IFNα-induced ISGylation and ubiquitination. While these findings appear to identify a novel crosstalk between different UBL modifications, they also indicate that care has to be taken when interpreting future proteomic experiments using ectopically expressed SUMO3, and potentially other SUMO paralogs (as used in the examples below), because of possible impacts on the basal functioning of the IFN system.

### 4.2. SUMO Proteomics during Herpes Simplex Virus 1 (HSV-1) Infection

The SUMO system is an important contributor to intrinsic (i.e., non-inducible and non-cytokine mediated) defenses against some virus infections, including HSV-1 [72,73]. HSV-1 expresses a regulatory protein, ICP0, that is able to induce the degradation of SUMOylated proteins, such as PML, thereby reducing the sensitivity of HSV-1 to inhibition by IFN [74]. To understand this system more, Sloan et al. investigated changes to the SUMO2 sub-proteome in response to infection with HSV-1 [37]. Sloan and colleagues showed that infection of hepatocytes with HSV-1 results in reduced global cellular SUMOylation, which was particularly apparent at later times post infection (10 h) and also occurred in SUMO2-overexpressing cells [37]. For the system-wide identification of SUMOylation changes to target proteins, HepaRG cells stably expressing HA-His-SUMO2 were grown in SILAC medium, infected with HSV-1 for 12 h, and both total protein and Ni-NTA-purified proteins were analyzed to determine infection-induced changes to total protein abundance and SUMOylated proteins, respectively. An important aspect of this work was the separation of fractions by SDS-PAGE prior to MS, which allowed gel slices to be analyzed individually, increasing the ‘depth’ of protein identification and allowing validation of SUMOylation by assessing the difference between estimated molecular mass in total cell lysates and apparent mass following His-SUMO2 purification (known as ‘slice-by-slice’ analysis). Of the 877 SUMOylated proteins identified, 260 changed in abundance upon HSV-1 infection, with a general trend towards loss of SUMOylated proteins. Strikingly, a large proportion of SUMOylated proteins that altered during infection were associated with transcriptional regulation or chromatin related pathways, and loss of many (but not all) SUMOylated targets was associated with the function of the viral ICP0 protein. Notable among the identified SUMOylated targets degraded during HSV-1 infection was the poorly characterized MORC3 protein, which was subsequently shown to harbor antiviral activity against HSV-1 and human cytomegalovirus by aiding the recruitment of PML nuclear body components to incoming viral genomes [75]. Thus, the application of unbiased SUMO2 proteomics during HSV-1 infection directly led to the identification of a new component of antiviral immunity against an important class of viruses. While this SUMO2 proteomic dataset lacks site-specific information due to the technology employed at the time, future studies using this resource will no doubt uncover additional novel insights into the interplay between DNA viruses and antiviral immunity.

### 4.3. SUMO Proteomics during Influenza Virus Infection

Our laboratory and others have shown that infection with influenza A virus (IAV) results in a global increase in cellular SUMOylation in various cell lines, while no substantial differences in the abundance of proteins of the SUMOylation machinery (UBC9, SAE1/2) were detected [33,76]. Furthermore, IAV infection results in an increase in SUMO-conjugates, but a depletion of free, unconjugated SUMO1 and SUMO2/3 [33]. The increased SUMOylation response was specific to nuclear-replicating RNA viruses, but not other cytoplasmic-replicating RNA virus infections, and was dependent on an active viral RNA polymerase [33]. To identify and quantify human and viral proteins that change in SUMO1 or SUMO2 modification status during IAV infection of human lung epithelial cells, our laboratory has used a TAP-tag based purification system, combined with SILAC proteomics [33]. The two SUMO sub-proteomes identified with this system were remarkably similar, which may suggest that the overexpression approach led to loss of SUMO paralog target specificity. Furthermore, site-specific information was not obtained due to the technology employed. Nevertheless, many bona fide SUMO substrates were identified to increase in SUMOylation during IAV infection, and these included typical SUMO target proteins involved in chromatin remodeling, DNA damage repair, transcription and RNA quality control. The proteomic analyses also uncovered that there was a widespread loss of SUMO from many substrates during infection, which was surprising given that the overall levels of SUMOylation appeared to increase during IAV infection when assayed by western blot. This discrepancy might be explained by the SUMOylation of viral proteins, such as NS1, M1 and NEP, that were identified as SUMO targets in this study and which are abundantly expressed during infection. Notable among the host proteins modified by SUMO during IAV infection was ANP32B, which (together with ANP32A) has subsequently been revealed to be a critical host factor for IAV polymerase activity, and a host barrier to viral cross-species transmission [77,78,79], although the precise role of SUMOylation in such processes remains to be clarified [80]. Furthermore, a large fraction of surveyed host proteins that increased in SUMOylation during IAV infection exhibited antiviral activity when screened functionally [33], including members of the PAF1 complex [81], possibly linking SUMOylation changes during IAV infections to the stimulation of innate antiviral immunity.

Additional SUMO proteomic studies on cells infected with influenza B virus, or a genetically-modified mutant IAV lacking its major IFN-antagonist protein, revealed that the widespread loss of SUMOylated proteins during IAV infection is generally conserved during these other virus infections [36]. While the complement of proteins that increased in SUMOylation status during influenza A and B virus infections was also similar, it was notable that infection with the IFN-stimulating mutant IAV led to a SUMOylation increase in only a small, yet distinct, set of substrates that included ISG15, an anti-influenza virus UBL [82]. The functional significance of ISG15 as a potential target of SUMOylation during viral infection has not been explored further, but may warrant attention. This finding is particularly intriguing given the reported cross-talk between SUMOylation and ISGylation recently described by others [34].

**Figure 6:**
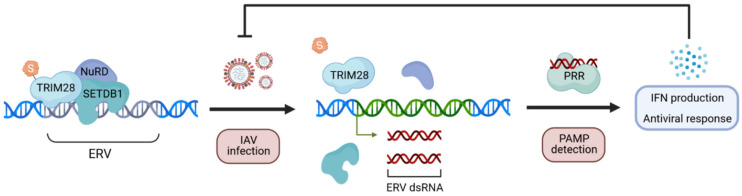
Example of how SUMO proteomics has identified a new type of innate antiviral response. Model of the infection-triggered TRIM28 SUMO-switch identified by proteomics that leads to increased antiviral responses. Influenza A virus (IAV) infection causes loss of SUMOylated TRIM28, destabilizing a multi-protein transcriptional repression complex and permitting endogenous retroelement (e.g., endogenous retrovirus, ERV) release. ERV expression can lead to the formation of endogenous ‘self’ double-stranded (ds) RNA that may be sensed by cellular Pattern Recognition Receptors (PRRs) that normally detect exogenous Pathogen-Associated Molecular Patterns (PAMPs), such as viral dsRNA, and trigger antiviral immunity via IFN production.

A striking observation that came from re-analysis of influenza virus-triggered changes to the host SUMOylated sub-proteome is related to the loss of SUMOylated TRIM28 during infection. TRIM28 is a known SUMO E3 ligase that acts as a transcriptional co-repressor molecule, together with several other factors (most importantly SUMO), to tightly and specifically silence endogenous retroelement (e.g., endogenous retrovirus; ERV) expression [83,84,85,86,87]. This mechanism is essential to prevent aberrant uncontrolled expression of immunostimulatory ERV nucleic acids, which might otherwise stimulate IFN-mediated autoimmune reactions [88,89,90]. It appears that this cellular safeguard system may also have been repurposed by the host in order to aid in transient defenses against invading viruses: infection-triggered loss of SUMOylated TRIM28 (a putative ‘SUMO-switch’) leads to derepression of immunostimulatory ERVs and the potentiation of IFN-mediated immunity [36] (Figure 6). This is yet another example of how large-scale unbiased SUMO proteomic screens in the context of viral infections have helped to uncover new types of innate antiviral immune responses. Future work with the very latest SUMO proteomic technologies will no doubt be critical to expand on these findings.

## 5. Concluding Remarks

Recent advances in MS-based technologies have facilitated the analysis of global changes in posttranslational modifications. Several novel methods have been established that enable the identification of site-specific SUMOylation, leading to the identification of thousands of SUMO targets. However, each of these technologies has certain advantages and disadvantages, with one major caveat being the dependence on ectopic expression of epitope-tagged SUMO and SUMO mutants. Overexpression might also overwhelm specificity in the SUMO machinery, resulting in SUMOylation of unnatural SUMO targets, unexpected artefacts, and detrimental changes in signal transduction. Therefore future research should focus on the establishment of physiologically relevant approaches, such as endogenous tagging of SUMO paralogs. For example, knock-in of His6-HA-tagged SUMO1 in mice seems to be well tolerated, and such mice showed no overt phenotypic abnormalities, yet might prove to be a useful tool [50]. In addition to identification of SUMO targets, characterization of the SUMO interactome will of course be of considerable interest, and a novel technique (SUMO-ID) was recently developed to tackle this challenge [68].

SUMOylation has been shown to be involved in many aspects of virus infection and innate immune responses, having both positive and negative effects. Current research interests have been dedicated to the dissection of global SUMOylation changes and determination of SUMO targets in the context of infection and IFN responses. A number of studies have therefore employed SUMO proteomics leading to the discovery of novel concepts in innate immunity [33,34,35,36,37]. However, many factors that had been previously described in small-scale studies were not detected with these methods. This might be owing to the experimental setup (nature of the stimulus, time points, cell lines, SUMO proteomics strategy), as, for example, there were no studies yet analyzing global SUMOylation after short times of infection or direct stimulation with TLR/RLR agonists that might be necessary to identify viral sensors or downstream signaling components as SUMO targets. Nevertheless, the datasets generated in these studies are undoubtedly opening avenues for further research.

Though a great deal of research has been devoted to SUMOylation and infection, many challenges and open questions remain. With the identification of SUMO substrates, SUMOylation sites, and the SUMO interactome, the challenge remains how to analyze the specific consequences of SUMOylation on its targets and in the wider context of the pathogen being studied. It further remains unclear how global changes in SUMOylation are triggered in the context of innate immune responses (e.g., cellular stress or direct induction by viral proteins). Delineating the molecular mechanisms of SUMO pathways might therefore open up novel opportunities for therapeutic interventions. For example, viruses that depend on SUMO-related mechanisms for replication could be inhibited by specific targeting of required SUMO-related host proteins. The observation that absence of SUMO2/3 results in a spontaneous IFN signature also suggests that the SUMOylation machinery might be a relevant target for autoimmune or auto-inflammatory therapeutics.
